# The value of innovation under value-based pricing

**DOI:** 10.3402/jmahp.v4.30754

**Published:** 2016-04-07

**Authors:** Santiago G. Moreno, Joshua A. Ray

**Affiliations:** Global Pricing and Market Access Department, F. Hoffmann-La Roche Ltd, Basel, Switzerland

**Keywords:** cost-effectiveness analysis, dynamic cost-effectiveness analysis, value-based pricing, innovation

## Abstract

**Objective:**

The role of cost-effectiveness analysis (CEA) in incentivizing innovation is controversial. Critics of CEA argue that its use for pricing purposes disregards the ‘value of innovation’ reflected in new drug development, whereas supporters of CEA highlight that the value of innovation is already accounted for. Our objective in this article is to outline the limitations of the conventional CEA approach, while proposing an alternative method of evaluation that captures the value of innovation more accurately.

**Method:**

The adoption of a new drug benefits present and future patients (with cost implications) for as long as the drug is part of clinical practice. Incidence patients and off-patent prices are identified as two key missing features preventing the conventional CEA approach from capturing 1) benefit to future patients and 2) future savings from off-patent prices. The proposed CEA approach incorporates these two features to derive the total lifetime value of an innovative drug (i.e., the value of innovation).

**Results:**

The conventional CEA approach tends to underestimate the value of innovative drugs by disregarding the benefit to future patients and savings from off-patent prices. As a result, innovative drugs are underpriced, only allowing manufacturers to capture approximately 15% of the total value of innovation during the patent protection period. In addition to including the incidence population and off-patent price, the alternative approach proposes pricing new drugs by first negotiating the share of value of innovation to be appropriated by the manufacturer (>15%?) and payer (<85%?), in order to then identify the drug price that satisfies this condition.

**Conclusion:**

We argue for a modification to the conventional CEA approach that integrates the total lifetime value of innovative drugs into CEA, by taking into account off-patent pricing and future patients. The proposed approach derives a price that allows manufacturers to capture an agreed share of this value, thereby incentivizing innovation, while supporting health-care systems to pursue dynamic allocative efficiency. However, the long-term sustainability of health-care systems must be assessed before this proposal is adopted by policy makers.

Producing innovative drugs is becoming ever more expensive. This situation is a reflection of the difficulty associated with their discovery and development. Manufacturers’ criticisms of current drug pricing are that this effort is not reflected in the price. This brings into question the future viability of manufacturers and the medical research that they sponsor. At the same time, health-care systems are under increasing pressure to efficiently allocate resources and obtain the most value for their investment. An example of this is the National Health System (NHS), which provides the majority of health care in the UK. In order to ensure the long-term sustainability of the NHS, spending is allocated to technologies that maximize population health given the budget constraints. The challenge is identifying a method that allows health-care systems such as the NHS to incentivize innovation while supporting allocative efficiency.

Setting the price of a new pharmaceutical product is a complex part of this process. A commonly used approach is to price products according to their value for patients, commonly referred to as *value-based pricing* (VBP) (1). The principle of VBP is to align the incentives for conducting research with the needs of patients, thereby generating valuable innovation (2). In cost-effectiveness analysis (CEA) VBP is established in relation to a CEA threshold. However, CEA is incapable of capturing a number of dimensions associated with innovative technologies (3). For example, health gains from innovative technologies that address an unmet need are valued (and thus priced) equal to health gains from less innovative technologies that address an already satisfied need (e.g., me-too drugs) (4). This is the case because CEA only rewards gains in clinical benefit, regardless of whether the gains come from an innovative technology or not. The result is a pricing and reimbursement decision that fails to adequately reward valuable innovation (5, 6).

The key to successfully addressing the suboptimal financial incentives reflected in the current pricing is to incorporate an innovation ‘value’ into the price of newly patented drugs. For this approach to move forward, however, manufacturers and policy makers must agree on how to measure the level of innovation of a new health technology. Currently, stakeholders participating in price negotiations articulate the level of innovativeness qualitatively (7), although some attempts have been made to value it quantitatively (8). Moreover, the concept of innovation cannot be directly translated into monetary terms or clinical benefit that can be later fed into a CEA. Previous attempts have favored raising the established CEA threshold, so as to reflect an inflated measure of willingness-to-pay for a quality-adjusted life-year (QALY), as opposed to reflecting the opportunity cost (8, 9). One example this approach has been used for is in the ‘end of life criteria’. This is specifically designed to reward new technologies that are able to extend the life of patients with a short life expectancy (10). Other initiatives, such as the 2014 ‘Value based assessment’, have failed to gain consensus between manufacturers, health-care providers, and other stakeholders (11, 12).

A major hurdle to reaching consensus around the measurement of innovation is the lack of a standardized definition of *innovation* itself (3, 7, 13). Here, we adopt the uncontroversial definition used by Claxton et al. (3), where innovation is restricted to new technologies that are claimed to offer benefits. The value of innovation is therefore defined here as the benefit that a new health technology brings to all patients (present and future) for as long it remains relevant for clinical practice.

Our objective in this article is to firstly identify the shortcomings of the conventional CEA approach to VBP in capturing the total lifetime value of innovative pharmaceutical drugs (hereafter named the *value of innovation*). Secondly, we propose modifications to the conventional CEA approach without advocating for a change to the CEA threshold. The proposed modifications aim to inform VBP by addressing the question of how the value of innovation is shared between the manufacturer and society (represented by a publicly funded health-care system). For illustration of a jurisdiction where the conventional CEA is used to inform drug prices, we use the National Institute for Health and Care Excellence (NICE), the reimbursement authority of the NHS in England and Wales. The proposal is illustrated through two hypothetical CEA case studies.

This paper focuses on patentable pharmaceutical drugs, but the findings are equally applicable to patentable medical devices such as diagnostics. It should be noted that there are other sources of innovation relevant to health-care providers, including new ways to deliver services and new surgical procedures (3), but these are not patentable.

## The role of cost-effectiveness in incentivizing innovation

The NHS adopts new health technologies if they are believed to offer good value for money. Within the CEA framework, this means that the additional cost required to gain one QALY with a new technology must not exceed a certain threshold. This threshold represents the marginal efficiency of the mix of existing NHS technologies. However, many of these existing technologies are off-patent and therefore inexpensive. Consequently, these ‘older’ technologies are highly efficient at generating QALYs. Thus, the comparison between existing NHS technologies and new ones does not reward the extra effort needed to foster innovation, reflected in the growing cost of research and development (R&D) for bringing new medicines into the market. Since 1970, global R&D costs have increased from £125 million ($199 million) to £1.2 billion ($1.9 billion) in the 2000s (both in 2011 prices) (14). This cost reflects the fact that the ‘low-hanging fruits’ of medical research have already been picked and the remaining unmet need requires an even larger research and financial effort. This current landscape leads manufacturers to raise concerns around the sustainability of medical research if ‘sufficient’ financial reward cannot be anticipated (15).

Allocation decisions based on comparing the incremental cost effectiveness ratio (ICER) of new technologies with a CEA threshold are intended to promote allocative efficiency of existent NHS resources. This has implications beyond the present day into future NHS efficiency because the adoption of new cost-effective technologies tends to displace less cost-effective technologies available in the NHS. In the long run, it is argued that this will improve NHS productivity, pulling down the CEA threshold even if the budget is kept constant over time (3).

It is therefore important to acknowledge that drug prices change over time and the effect such change has on NHS productivity. For example, the drop in drug prices due to generic entry represents a significant transfer of value from the industry to the NHS (16). The impact of this transfer of value is exemplified by statins, which, according to Claxton et al. (3), ‘were cost-effective when introduced and improved the productivity of the NHS (tending to reduce the threshold). They then became much cheaper on generic entry dramatically increasing productivity (also tending to reduce the threshold further)’. Indeed, according to the latest published information, between 2004/2005 and 2010/2011, the NHS enjoyed an 8% increase in productivity, which was partially due to falling drug prices (17).

In summary, while valuable innovation is becoming ever more challenging and expensive to develop, the conventional VBP approach employed by the NHS does not reward the added effort but instead tends to diminish future financial incentives as a result of a falling CEA threshold.

## Method

To capture the value brought by an innovative drug requires consideration of its entire market lifetime, specifically, the period over which the technology is part of clinical practice and can therefore generate the expected health benefits. The conventional CEA approach undervalues innovative technologies because it disregards key features of the drug lifetime, including benefits to future (incidence) patients and the savings that off-patent prices bring to the NHS. Recently, these features have been successfully implemented in ‘dynamic’ CEA (18–22). The typical features of dynamic CEAs (above and beyond those from the conventional CEA) relate to time-dependent variations of the following:

- *Drug prices* (23): price erosion and off-patent price
- *Size of the population treated* (24): coverage level, market penetration and disease incidence

These features can be categorized as either *exogenous* or *endogenous* to the NHS, where *endogenous* refers to features under the control or influence of the NHS. Endogenous features include the periodic price cuts that cause price erosion during patent protection (18, 25), as well as the level of coverage and market penetration. Adjusting the ICER to account for historical trends in any endogenous features is of questionable value, because it could trigger an escalation in restrictions (e.g., an upsurge of price erosion). Hence, the approach proposed here excludes the endogenous features and incorporates the exogenous ones. Specifically, the proposed approach adds two features to the conventional CEA approach: incident patient population and a constant off-patent price from the time of patent expiry.

The conventional CEA approach makes the somewhat naïve assumption that drug prices remain unchanged even after patent expiry. This assumption ignores the predictable arrival of cheap generics/biosimilars at patent expiry (26). In order to account for this transfer of value from the industry to the NHS, the proposed VBP approach requires inputting the time to patent expiry and the anticipated off-patent price, which applies to patients treated after patent expiry. Then, the proposed VBP approach works by identifying the on-patent price, which jointly with the off-patent price makes the drug cost-effective and therefore a worthy investment for the NHS. The dynamic CEA perspective adopted here promotes dynamic efficiency in the allocation of present and future NHS resources (that maximize the surplus of present and future patients). In contrast, the conventional VBP approach identifies the price that makes the drug cost-effective for a typical patient at the time of product launch. This conventional CEA perspective induces immediate (static) efficiency in the allocation of NHS resources (by maximizing the surplus of prevalent patients given existent NHS resources).

A different scale to the cost-effectiveness ratio is needed to quantify the total lifetime value of an innovative drug (value of innovation). The advantage of the incremental net health benefit (INHB) scale (27) is that it unifies the two CEA dimensions (health benefits and costs) into one (see Supplementary file) (27). The cumulative INHB (cINHB) function can be conceptualized as the net present value (NPV) function commonly used to forecast the profitability of an investment. The associated decision rule is to invest in the new technology if the cINHB≥0. The cINHB function captures the time-dependency of health benefits and costs, making it ideally suited for exploring the fluctuation in the value of a health technology over time (28). This allows the NHS to view the new technology as an investment and predict how long it will take to pay it off (2). That is, the NHS can predict the moment when the investment in a specific technology will break even (cINHB=0) to then start capitalizing the positive cINHB thereafter. From the NHS perspective, the sooner the ‘break-even’ occurs, the less risky the investment (2). The pattern of a typical cINHB function begins with negative values because the NHS accrues the drug costs before any health benefits are realized. The trend starts reversing with the realization of health benefits in the form of QALY gains. The break-even point (cINHB=0) occurs when the QALY gains fully offset the added costs. The cumulative INHB turns positive once the QALY gains exceed the added costs and, more importantly, it remains positive for as long the technology is relevant for clinical practice (2).

The estimation of the ‘total’ value of a technology requires a judgment about the time horizon over which the technology will be utilized (29). This technology time horizon represents its market lifetime until it becomes obsolete for clinical practice. Based on historical data of drug usage in England, the average lifetime of a drug in the market is around 33 years (18). Hence, the total lifetime value of innovation is estimated as the cumulative INHB 33 years after market launch.

## Applied examples

**Fig. 1:**
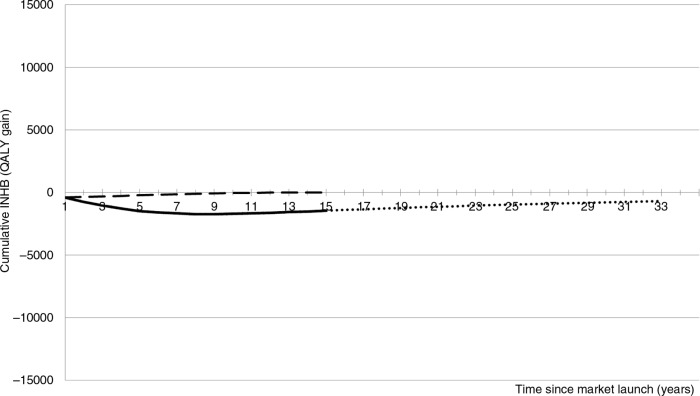
Dashed line: cumulative incremental net health benefit (cINHB) along the patient time horizon under the conventional cost-effectiveness analysis (CEA) approach. Solid line: cINHB along the patient time horizon, adding incidence cohorts to the conventional CEA approach. Dotted line: cINHB extension of the solid line covering the drug lifetime (without accounting for the off-patent price).

**Fig. 2:**
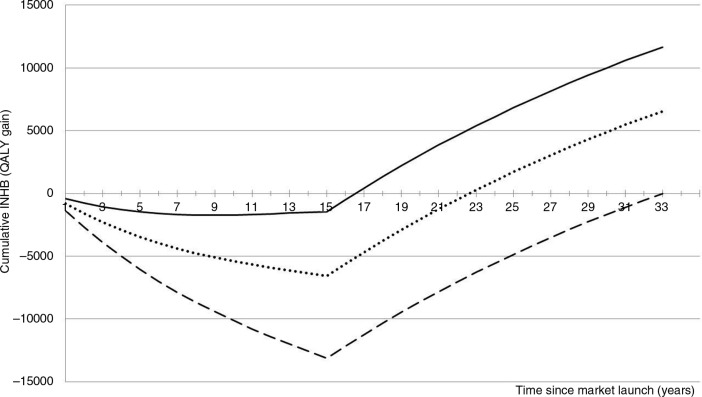
cINHB along the drug lifetime (in the chronic disease setting) accounting for the off-patent price. Solid line: cINHB under the conventional CEA approach. Dotted line: cINHB under the proposed CEA approach. Dashed line: cINHB under a hypothetical scenario where the manufacturer captures 100% of the value of innovation during patent protection.

**Fig. 3:**
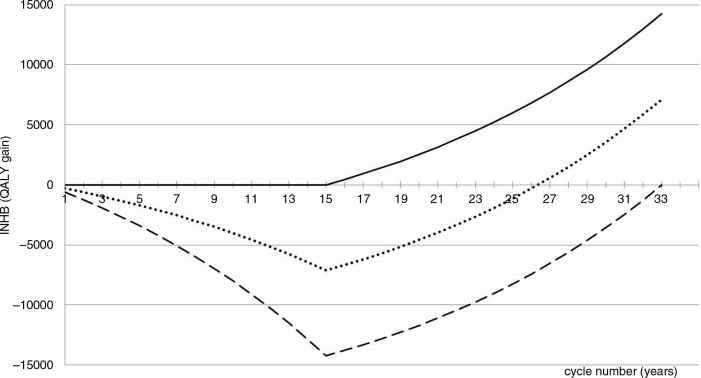
cINHB along the drug lifetime (in the acute disease setting) accounting for the off-patent price. Solid line: cINHB under the conventional CEA approach. Dotted line: cINHB function under the proposed CEA approach. Dashed line: cINHB under a hypothetical scenario where the manufacturer captures 100% of the value of innovation during patent protection.

Two theoretical CEA case studies are used for illustrating the impact on the drug price from adopting the proposed VBP approach compared to the conventional one. They are intended to represent the treatment of a disease in chronic and acute settings in a simplistic manner (Table 1). Figures 1 and 2 provide a step-by-step illustration of the implementation of the proposed VBP approach in a chronic disease setting, whereas the acute setting is illustrated in Fig. 3.

**Table 1:** Description of two CEA case studies

Features common to both case studies	CEA threshold=£30,000/QALY gain. Patients are diagnosed at the age of 50 years. QALYs and costs are both discounted at an annual rate of 3.5%. Patent expires 15 years after market launch. The off-patent price is 25% of the on-patent price.
Chronic disease setting: anticancer treatment	The incidence of cancer remains constant with 1,300 new patients every year. Both the experimental and standard of care (SoC) treatments are administered for 1 year. After that, all patients progress to the next treatment line due to progression of the disease. During the 1-year treatment, patients experience a utility value of 0.9 with the experimental oncology drug, while patients under the SoC experience a utility of 0.7. The utility value is 0.7 for all patients after the 1-year treatment. The experimental oncology drug extends life by 1 year, making the life expectancy shift from a maximum of 14 years to a maximum of 15 years (patient time horizon). One-year treatment under SoC costs £20,000 per patient, and subsequent lines of treatment cost £10,000 per patient annually until death.
Acute disease setting: antibiotic treatment for antimicrobial resistance	The incidence in the first year is 600 patients, and it is assumed to grow by a constant 10% annually to represent the wide spread of multidrug-resistant infections across the general population (30). Patients experience a utility value of 0.7 with the SoC during 1 year (patient time horizon). The experimental antibiotic immediately restores the utility to 0.9. No gain in survival is considered. One-year treatment under SoC costs £20,000 per patient.

QALY, quality-adjusted life-year.

### Figure 1: Conventional approach to CEA

The time horizon of a conventional CEA refers to the patient, not the technology. Guidance from NICE (31) specifies that the ‘patient time horizon’ should be long enough to capture all the differences in benefits between the new technology and its comparator. Therefore, a relatively short time horizon is required to model the CEA of an acute infection without long-term sequelae. Likewise, a lifetime horizon is required when the benefits of the new technology persist for the remainder of the patient's life.

According to VBP, as conventionally applied (dashed line), the maximum price that the manufacturer can charge the NHS for the new drug is given by constraining the break-even point (cINHB=0) to occur at the end of the patient time horizon (15 years in the cancer example). The price of the new oncology drug that satisfies this condition is £35,000 per patient/year (£15,000 in excess of the comparator's price of £20,000).

### Figure 1 continuation: Adding incidence cohorts to the population modeled

The conventional CEA approach models an inception cohort without consideration to subsequent incidence cohorts. In line with other authors, we advocate modeling incidence cohorts because future patients will also benefit from the new drug (18, 32). Accounting for the entire patient population offers the additional advantage of internal consistency between the CEA and budget impact estimates, which is lacking under the conventional CEA approach (33).

The solid line in Fig. 1 includes the incidence cohorts. Fifteen years after market launch, this new drug is not a valuable investment for the NHS because it will have displaced more QALYs than it produces (cINHB<0).

### Figure 1 continuation: The patient versus the technology-based time horizon

The time horizon for the technology is different to that of the patient. To capture the total value of an innovative technology requires consideration of the benefit it will bring to all patients during its entire market lifetime (dotted line). The total value of innovation is given by the cumulative INHB function at Year 33 (18) (i.e., the total lifetime value of innovation=−700 QALYs). The negative value of the dotted line indicates that the new drug (£35,000 per patient/year) will have displaced more QALYs than it produces during its lifetime. Hence, the new drug is not a worthy investment for the NHS.

### Figure 2: Patent expiry and arrival of generic/biosimilar

Figure 2 illustrates the transfer of value from the industry to the NHS resulting from the arrival of generics/biosimilars at patent expiry. In this example, the INHB losses that were accrued during the initial 17 years are more than offset by later gains, turning the new drug into a worthy investment for the NHS. The total value of innovation is 12,000 QALYs 33 years after market launch (solid line).

The solid line exemplifies how the value of innovation is currently (and perhaps inadvertently) being shared between manufacturers and the NHS. In this case study, the drug price of £35,000 per patient/year guarantees that the manufacturer will capture 12% of the total value of innovation by the time of patent expiry (15 years). The 12% share is estimated as follows:

$$=\frac{Cumulative\;INHB\;at\;patent\;expiry\;with\;an\;on-patent\;price\;of\;₤\text{35},\text{000}}{Cumulative\;INHB\;at\;patent\;expiry\;with\;an\;on-patent\;price\;that\;captures\;\text{100}\%\;of\;value\;during\;patent\;protection}$$

The denominator represents a hypothetical scenario wherethe manufacturer captures 100% of the total lifetime value of innovation during patent protection. Theoretically, this can be achieved if the manufacturer promises to sell the drug at a negligible price (e.g., production cost) after patent expiry, while constraining cINHB=0 at year 33. The dashed line represents this scenario, where the on-patent price of £57,500 per patient/year (=37,500+20,000) is set to capture 100% of the total lifetime value of innovation during patent protection.

### Figure 2 continuation: The proposed VBP approach: sharing the value of innovation

The dashed line makes the unrealistic assumption that the health-care system is willing to let the manufacturer capture the whole value of innovation during the patent protection period. A more socially responsible proposal is to share it. In this case study, we apply a 50–50% split to illustrate the workings of the proposed approach. In addition, we assume an off-patent price of zero, representing a negligible cost of production after patent expiry. Note that the lower the off-patent price the higher the on-patent price.

The proposed VBP approach works by identifying the on-patent price (alongside the anticipated off-patent price) that guarantees that the manufacturer will capture 50% of the total value of innovation before the patent expires. The dotted line depicts the resulting cumulative INHB function with a drug price of £45,000 per patient/year (=25,000+20,000). This compares to 12% with a drug price of £35,000 (=15,000+20,000) under the conventional VBP approach.

### Figure 3: Case study in the acute disease setting

Under the conventional VBP approach (solid line) the incremental on-patent price of the new antibiotic is £6,000 per patient in excess of the competitor's price £3,000. This price makes the new antibiotic a very worthy investment for the NHS because it pays off from the very first year. As a result, the manufacturer is unable to capture any share of the total value of innovation (14,000 QALYs). On the opposite extreme (dashed line), the on-patent price captures 100% of the value of innovation. Under the proposed VBP approach (dotted line), a price for the new antibiotic of £24,000 per patient (=21,000+3,000) guarantees that 50% of the total value of innovation is captured by the manufacturer during the patent protection period.

## Discussion

This article contributes to the much-needed debate about the role of CEA in incentivizing innovation. Specifically, the debate centers on the currently used conventional CEA for reimbursement decisions under a strict CEA threshold, and whether neglecting the value of innovation prevents VBP from capturing the inherent value of newly patented health technologies, leading to suboptimal incentives for future research.

The principle of VBP is intended to align the incentives for innovation with the needs of patients (2). However, as conventionally applied, VBP grants me-too drugs the same price as the originator if the two are clinically comparable (4), disincentivizing the development of truly innovative technologies (34). The integration of the value of innovation into VBP is intended to incentivize the development of truly innovative technologies by granting prices that better reflect their true lifetime value. Indeed, the proposed VBP approach will grant lower prices to me-too drugs compared to truly innovative medicines. This is possible because the dynamic CEA takes into account the cost savings generated by the earlier patent expiry of the originator.

Another important feature of the proposed VBP approach is that it accounts for the benefits that the technology will bring to both present and future patients. In this sense, drugs aimed at treating diseases with a growing incidence rate, like antibiotics for antimicrobial resistance, are granted significantly higher prices compared to the conventional VBP approach. This supports the belief that the conventional VBP approach is falling short when valuing such technologies because it disregards the benefit to future patients (35–37). This undervaluation is a direct consequence of applying a static perspective when making allocation decisions (that maximize the surplus of prevalent patients given existent NHS resources). In contrast, the proposed VBP approach allows the NHS to promote dynamic efficiency in the allocation of present and future NHS resources (that maximize the surplus of present and future patients). The dynamic perspective is preferred because it aligns the incentives for innovation with the needs of both present and future patients. It is also preferred because it allows the allocation of future NHS resources efficiently. This is achieved by taking into account the cost savings to be generated by the earlier patent expiry of the older drug (comparator) when setting the price of the new drug. This strategy is in line with a dynamic perspective and helps minimize the up-front capitalization of benefits unrealized due to premature displacement. By accounting for the future off-patent price of the displaced drug, the price of the newer drug is pushed down. This is particularly impactful when the displaced drug is close to patent expiry and the price drop is expected to be substantial. Under such a scenario, the newer and better drug could be potentially priced lower that the older drug. Noticeably, this factor is not considered by the conventional approach; as a result, the newer and better drugs are always priced higher than older drugs, regardless of the time to patent expiry of the comparator. This questions the ability of the conventional approach to allocate future NHS resources efficiently.

The proposed VBP approach offers the opportunity to explicitly address the question of how innovation ought to be incentivized (38, 39). Specifically, price negotiations can benefit from a clear understanding on how the choice of price affects the share of value captured by manufacturers and health-care systems. Ultimately, the chosen (on-patent) price guarantees the appropriation by the manufacturer of the agreed share during the patent protection period. Our two case studies apply a 50–50% split for illustrative purposes. This compares to 15–85% (in favor of the health-care system) under the conventional VBP approach (38). Note that the larger the share captured by the manufacturer, the higher the drug price. To determine this split value, we recommend eliciting the share of value that society is willing to forgo in order to incentivize innovation.

NICE is taken as example of a reimbursement authority where CEA is routinely used to inform drug prices. The generalizability of the proposed VBP approach to other jurisdictions (including low- and middle-income countries) is possible by adapting the value of the following model parameters to the local setting: 1) local cost-effectiveness threshold; 2) percentage share of value that this jurisdiction is willing to forgo to incentivize innovation; 3) local disease incidence and prevalence; 4) average lifetime of a drug in local market; 5) local off-patent price of the new drug under evaluation and the comparator. The last four model parameters are unique to the proposed VBP approach. They would benefit from further research to reduce uncertainty around their true values and learn how much they vary, for example, by therapeutic area or jurisdiction.

Under the proposed VBP approach, a low off-patent price allows the manufacturer to obtain a higher on-patent price. The drop in price after patent expiry can be achieved by switching all prescribing to generics/biosimilars (assuming that the market is competitive) or by cutting the price of the originator. One provocative idea to help reduce uncertainty around the future off-patent price is to negotiate it at the time of market launch and to guarantee its sale for the same (inflation-adjusted) price for as long as the drug remains relevant for clinical practice. Under this scenario, a market access strategy devised to elude fierce competition after patent expiry is to accrue as much value as possible during the patent protection period. This can be achieved if the manufacturer offers an off-patent price equal to the production cost. The negotiation of off-patent prices at the time of market launch has three long-term consequences:

1. The progressive loss of viability of the generic/biosimilar industry, particularly as the off-patent prices of originators are negotiated downwards.
2. Assuming that the (financial) resources of the generic/biosimilar industry remain invested in health research, they will generate valuable innovation able to fulfill the still existing unmet medical need.
3. The industry as a whole is strongly incentivized to develop innovative technologies because there is limited revenue to be generated from off-patent products.

In line with Lundin and Ramsberg (40), we acknowledge that accounting for the value of innovation will increase spending on innovative technologies. However, the impact on health-care budgets can be partially mitigated by restraining the prices of less innovative technologies (e.g., me-too drugs) as well as from the off-patent prices of originators.

## Conclusion

The methods employed here illustrate how the value of innovation can be integrated into CEA to derive an appropriate price for innovative drugs under VBP. This is intended to incentivize innovation while supporting health-care systems’ pursuit of dynamic allocative efficiency.

Last, this VBP proposal would benefit from more experience in order to advance understanding of the feasibility and implications of its adoption by policy makers and manufacturers, with a special focus on budget impact and subsequent NHS sustainability. To gain more experience with the proposed VBP approach, we recommend its implementation as an additional scenario to the conventional CEA and then reporting the CEA findings comparing the results of each approach.

## Supplementary Material

The value of innovation under value-based pricing

## References

[1] Gregson N, Sparrowhawk K, Mauskopf J, Paul J (2005). Pricing medicines: Theory and practice, challenges and opportunities. Nat Rev Drug Discov.

[2] Claxton K, Sculpher MJ, Carroll S (2011). Value-based pricing for pharmaceuticals: Its role, specification, and prospects in a newly devolved NHS.

[3] Claxton K, Longo R, Longworth L, McCabe C, Wailoo A (2009). The value of innovation. Decision support unit.

[4] Claxton K, Lindsay AB, Buxton MJ, Culyer AJ, McCabe C, Walker S (2008). Value based pricing for NHS drugs: An opportunity not to be missed?. BMJ.

[5] Healy P, Pugatch M (2012). Capturing value: Why dynamic efficiency should be considered in the pricing and reimbursement of medicines.

[6] Rejon-Parrilla JC, Hernandez-Villafuerte K, Shah K, Mestre-Ferrandiz J, Garrison L, Towse A (2014). The expanding value footprint of oncology treatments.

[7] Henshall C, Schuller T (2013). Health technology assessment, value-based decision making, and innovation. Int J Technol Assess Health Care.

[8] Thokala P, Duenas A (2012). Multiple criteria decision analysis for health technology assessment. Value Health.

[9] Claxton K, Martin S, Soares MO, Rice N, Spackman E, Hinde S (2013). Methods for the estimation of the NICE cost effectiveness threshold.

[10] Collins M, Latimer N (2013). NICE's end of life decision making scheme: Impact on population health. BMJ.

[11] National Institute for Health and Care Excellence (NICE) (2014). Value based assessment methods consultation document.

[12] National Institute for Health and Care Excellence (NICE) (2014). NICE calls for a new approach to managing the entry of drugs into the NHS. NICE.

[13] Canadian Agency for Drugs and Technologies in Health (CADTH) (2010). The economic value of innovative health technologies.

[14] Mestre-Ferrandiz J, Sussex J, Towse A (2012). The R&D cost of a new medicine.

[15] Refoios Camejo R, McGrath C, Miraldo M, Rutten F (2013). The determinants of cost-effectiveness potential: An historical perspective on lipid-lowering therapies. Pharmacoeconomics.

[16] McKellar M, Matthew F, Haiden H, Chernew M (2012). The value of patent expiration. Forum Health Econ Policy.

[17] Bojke C, Castelli A, Grasic K, Street A, Ward P (2013). University of York. NHS productivity from 2004/5 to 2010/11.

[18] Hoyle M (2011). Accounting for the drug life cycle and future drug prices in cost-effectiveness analysis. Pharmacoeconomics.

[19] Garrison J, Veenstra DL (2009). The economic value of innovative treatments over the product life cycle: The case of targeted trastuzumab therapy for breast cancer. Value Health.

[20] Garrison J (2010). Rewarding value creation to promote innovation in oncology: The importance of considering the global product life cycle. Oncologist.

[21] Grabner M, Johnson W, Abdulhalim AM, Kuznik A, Mullins CD (2011). The value of Atorvastatin over the product life cycle in the United States. Clin Ther.

[22] Lu Y, Penrod JR, Sood N, Woodby S, Philipson T (2012). Dynamic cost-effectiveness of oncology drugs. Am J Manag Care.

[23] Millier A, Briquet B, Aballea S, Toumi M (2014). Should changes in drug price over time be considered in cost-effectiveness analyses? Value In Health.

[24] Hoyle M, Anderson R (2010). Whose costs and benefits? Why economic evaluations should simulate both prevalent and all future incident patient cohorts. Med Decis Mak.

[25] NHS (2013). The Pharmaceutical Price Regulation Scheme 2014.

[26] Palmer E Deep discounts allow Remicade biosimilar to grab 50% of Norway's market. FiercePharma 2015.

[27] Stinnett AA, Mullahy J (1998). Net health benefits: A new framework for the analysis of uncertainty in cost-effectiveness analysis. Med Decis Mak.

[28] McCabe C, Edlin R, Hall P (2013). Navigating time and uncertainty in health technology appraisal: Would a map help?. Pharmacoeconomics.

[29] Claxton K, Palmer S, Longworth L, Bojke L, Griffin S, McKenna C (2011). Uncertainty, evidence and irrecoverable costs: Informing approval, pricing and research decisions for health technologies.

[30] Walsh F (2014). Superbugs to kill ‘more than cancer’ by 2050.

[31] National Institute for Health and Care Excellence (NICE) (2013). Guide to the methods of technology appraisal.

[32] Ethgen O, Standaert B (2012). Population-versus cohort-based modelling approaches. Pharmacoeconomics.

[33] Mar J, Sainz-Ezkerra M, Miranda-Serrano E (2008). Calculation of prevalence with Markov models: Budget impact analysis of thrombolysis for stroke. Med Decis Mak.

[34] Pekarsky B (2010). Should financial incentives be used to differentially reward ‘me-too’ and innovative drugs?. Pharmacoeconomics.

[35] Smith R, Coast J (2013). The true cost of antimicrobial resistance. BMJ.

[36] White AR, Blaser M, Carrs O, Cassell G, Fishman N, Guidos R (2011). Effective antibacterials: At what cost? The economics of antibacterial resistance and its control. J Antimicrob Chemother.

[37] Scandlen-Finken L, Wertheimer A (2015). Incentivizing antibiotic research and development.

[38] Jena AB, Philipson TJ (2008). Cost-effectiveness analysis and innovation. J Health Econ.

[39] Jena AB, Philipson TJ (2013). Endogenous cost-effectiveness analysis and health care technology adoption. J Health Econ.

[40] Lundin D, Ramsberg J (2008). Dynamic cost-effectiveness: A more efficient reimbursement criterion. Forum Health Econ Policy.

[41] McCabe C (2009). What is cost-utility analysis.

