# Supplementary material for: The value of innovation under value-based pricing
Source: J Mark Access Health Policy. 2016 Apr 7;4:10.3402/jmahp.v4.30754. doi: 10.3402/jmahp.v4.30754 (PMC4826462; doi:10.3402/jmahp.v4.30754)
Supplement: The value of innovation under value-based pricing [file JMAHP-4-30754-s001.docx]

**Supplemental Materials – for online publication**

*(Reason for adding this appendix: To provide the definition of INHB to readers)*

**APPENDIX**

In a cost-utility analysis, the consequences of implementing a new technology are measured in terms of incremental costs and incremental QALYs (41). The Incremental Net Health Benefit (INHB) scale measures all benefits in the QALY scale (27) by transforming costs into QALYs using the CEA threshold as the conversion rate. For example, an incremental cost of £60,000 is valued as 2 QALYs when the CEA threshold is £30,000/QALY.

The INHB statistic is presented next:

INHB = ΔE – (ΔC / CEA threshold)

The decision rule applied in CEA is to implement the new health technology if the ICER≤CEA threshold. The INHB statistic is a linear transformation of the ICER. The corresponding decision rule is to implement the new health technology if the INHB≥0.

- When INHB<0 🡺 ICER>CEA threshold
- When INHB=0 🡺 ICER=CEA threshold
- When INHB>0 🡺 ICER<CEA threshold

Legends: ΔC: Incremental Costs; ΔE: Incremental QALYs; INHB: Incremental Net Health Benefit; ICER: Incremental Cost Effectiveness Ratio; CEA: Cost-Effectiveness Analysis; QALY: Quality-Adjusted Life-Year
